# *Bartonella* Neuroretinitis with Initial Seronegativity and an Absent Macular Star: A Case Report and Literature Review

**DOI:** 10.3390/tropicalmed9080186

**Published:** 2024-08-20

**Authors:** Jason Timothy Pan, Dayna Wei Wei Yong, Hazel Anne Lin

**Affiliations:** 1 Yong Loo Lin School of Medicine, National University of Singapore, Singapore 119077, Singapore; jason.pan@u.nus.edu; 2 Department of Ophthalmology, National University Hospital, 1E Kent Ridge Rd, Singapore 119228, Singapore; daynayong6@gmail.com

**Keywords:** *Bartonella henselae*, cat-scratch disease, neuroretinitis

## Abstract

Cat-scratch disease (CSD) is an infectious disease caused by *Bartonella henselae*, presenting with fever and lymphadenopathy following contact with felines. The ocular manifestations include neuroretinitis, characterised by optic nerve swelling and a macular star. Case Presentation: We discuss a case of neuroretinitis that presented atypically, without a macular star. There was an initial suspicion of *Bartonella*, but the serology was negative. Our patient was eventually empirically treated for infective neuroretinitis based on a positive contact history (recently scratched by one of his three pet cats). There was progression to a macular star upon serial dilated fundus examination, and the repeated serology one week after symptom onset showed rising titres, supporting a diagnosis of CSD. Conclusions: A judicious review of systems, repeat assays, serial dilated fundus examination, and early ophthalmic evaluation are useful in cases of suspected neuroretinitis, remaining an important differential in the evaluation of sudden-onset painless vision loss and unilateral disc swelling.

## 1. Background

Neuroretinitis is the inflammation of the anterior optic nerve and peripapillary retina, and it manifests clinically as optic disc swelling and pathognomonic macular star appearance. Causative agents vary and can be broadly classified into infective, inflammatory, and idiopathic aetiologies [1,2]. Globally, *Bartonella henselae* is the leading cause of infective neuroretinitis [3]. While the reported seroprevalence of *Bartonella henselae* in Singaporean cats is 47% [4], CSD remains uncommon locally and is not included in the routine workup for optic disc swelling. Additionally, very few cases of neuroretinitis have been reported in Singapore [5], and local data on *Bartonella* neuroretinitis are limited. To our knowledge, there have been no case reports of *Bartonella* neuroretinitis in Singapore.

## 2. Case Presentation

A 23-year-old Malay male was referred for sudden onset of painless, central blurring of vision in the right eye, with no subsequent progression of symptoms, no associated floaters or photopsia, no pain on eye movement, and no symptoms in the fellow eye. System review revealed a prodrome of headache, low-grade fever (37.7 °C), and painful cervical lymphadenopathy 2 days before the onset of visual symptoms, for which he was prescribed oral amoxicillin/clavulanic acid by his General Practitioner in view of a possible infection. There was no significant travel history, sexual history, or sick contacts.

**Figure 1:**
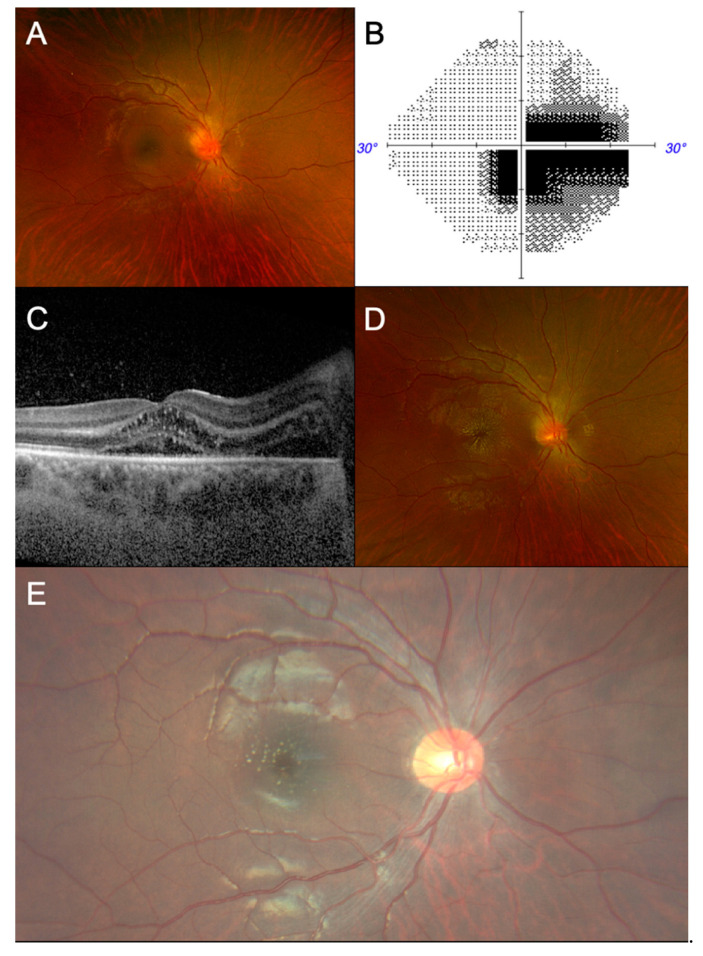
(**A**) From left to right, fundus photograph of the patient’s right eye at first presentation reveals hyperaemic optic disc swelling with macular oedema covering two disc diameters. (**B**) Static automatic perimetry shows a centrocecal scotoma in the right eye. (**C**) Optical coherence tomography of the right macula shows suggestion of intraretinal and subretinal fluid extending from the optic nerve and the presence of hard exudates in Henle’s layer. (**D**) Fundus photograph showing progression to the formation of a full macular star over the course of 1 week. (**E**) Fundus photograph after 2 weeks of treatment, showing resolution of optic disc and macular oedema, with residual hard exudates in the configuration of a resolving macular star.

On examination, his best corrected visual acuity (BCVA) was counting fingers at a distance of 1 meter using the right eye. The BCVA of the fellow eye was 6/6. There was a right, grade-1 relative afferent pupillary defect (RAPD) with marked red desaturation. Ishihara testing could not be performed in the right eye. The left eye’s optic nerve function was normal. A fundus examination of the right eye showed a diffusely swollen optic disc and macular oedema (Figure 1A). The examination of the left eye was normal. Left submandibular lymph nodes were noted. There was no associated cranial nerve deficit.

Blood investigations revealed leucocytosis (12.76 × 10^9^ cells/L), neutrophilia (9.29 × 10^9^/L), raised C-reactive protein (62.6 mg/L) and raised erythrocyte sedimentation rate (44 mm/hr). A pre-steroid workup was commenced and was unremarkable. Autoimmune antibodies (anti-aquaporin 4, anti-myelin oligodendrocyte glycoprotein, anti-nuclear antibody, anti-double-stranded DNA antibody) were negative. Initial *Bartonella* and *Borrelia* immunoglobulin G (IgG) titres to rule out neuroretinitis were negative (<1:64). Static automated perimetry revealed a centrocecal scotoma in the right eye (Figure 1B). Optical coherence tomography (OCT) of the right eye showed a suggestion of intraretinal fluid and subretinal fluid at the optic nerve extending to the macula (Figure 1C). There was right-disc leakage on fundus fluorescein angiography (FFA). Magnetic resonance imaging (MRI) of the brain, orbit, and anterior visual pathway with contrast and lumbar puncture were unremarkable. As all investigations were unremarkable, the patient was thus managed as a case of optic disc swelling for evaluation. He was monitored closely as an inpatient for the progression of signs and symptoms.

BCVA in the right eye improved spontaneously to 6/60-1 without treatment. Five days after symptom onset, a half-star of hard exudates appeared and progressed to macular star tracking temporally (Figure 1D). His history was revisited, and subsequent questioning revealed he had been recently scratched by one of his three pet cats. A provisional diagnosis of right neuroretinitis was made. In view of the prodromal symptoms, raised inflammatory markers, and history of cat exposure, he was treated for infective neuroretinitis, and started on oral doxycycline 100 mg twice a day for 2 weeks, with 10 mg of oral prednisolone once a day, tapered to 2.5 mg weekly thereafter.

In view of a possible false negative, *Bartonella henselae* IgG serology was repeated 1 week after and showed rising titres (1:128, equivocal). A subsequent review in the clinic 2 weeks later showed an improvement in the BCVA to 6/24-1, accompanying an improvement in the visual field defect and the resolution of disc swelling and macula oedema. The macula appeared flat, with hard exudates in the configuration of a resolving macular star (Figure 1E). The BCVA at the last review was 6/9-2 in the right eye. The final diagnosis was *Bartonella* neuroretinitis.

## 3. Discussion

In the appropriate clinical context, it is important to consider CSD in the evaluation of painless unilateral optic disc swelling, such as the presence of prodromal symptoms, lymphadenitis, a history of cat exposure, or a macular star [6], all of which were present in our patient, albeit after directed questioning and serial evaluation. The causes of unilateral optic disc swelling can be broadly classified anatomically into intracranial, retrobulbar and ocular causes, whereby each disease entity presents with a unique constellation of signs and symptoms (Table 1). Neuroretinitis involves both the optic nerve head and the retrobulbar optic nerve, and it can mimic the more prevalent optic neuritis [7], especially in the early stages, where only isolated optic disc swelling is seen. Thus, the differential diagnosis was kept broad to avoid missing potentially life-threatening aetiologies, which was reflected in the initial diagnostic workup.

A thorough evaluation for optic neuritis in the context of isolated disc swelling includes MRI neuroimaging, autoantibodies, and a lumbar puncture. Given the local prevalence data, serological testing for neuromyelitis optica spectrum disease (NMOSD) and myelin oligodendrocyte glycoprotein antibody-associated disease (MOGAD) is considered routine in optic neuritis investigation in Singapore [8]. The appearance of a macular star generally precludes the possibility of a demyelinating aetiology [9]. OCT is a non-invasive imaging modality that produces cross-sectional imaging to evaluate the retina, macula, and optic nerve head [10]. While conventional wisdom states that a macular star has to be present to reliably diagnose neuroretinitis, OCT was shown, in our case, to detect subretinal and intraretinal fluid before a macular star formed, which has been previously reported [11]. Thus, early evaluation by an ophthalmologist can help to potentially avoid unnecessary testing and streamline diagnosis. Neuroretinitis should be differentiated from other causes of optic disc oedema with a macular star, most commonly malignant hypertension, which usually presents bilaterally and is often mistaken for neuroretinitis [3].

Electrophysiology studies can aid in the evaluation of optic nerve disease. Pattern visual-evoked potentials (VEPs) typically show an increased latency and a decreased amplitude in demyelinating diseases such as optic neuritis [12], but they are typically very mild in neuroretinitis (i.e., mild reduction in P100 on pattern VEP without delay) [13]. Electroretinography (ERG) measures the functional integrity of the photoreceptors and bipolar cells, which are preserved in neuroretinitis, as it involves the ganglion cells and the optic nerve [14]. These investigations are time-consuming and were not prioritised in our patient in view of cost concerns and our low index of suspicion for a demyelinating aetiology after the initial unremarkable workup.

A serological diagnosis can be supportive and should include the most common infective aetiologies (i.e., *Bartonella* and *Borrelia* titres) where accessible. While other infectious agents have been described in other Asian cohorts (e.g., *Toxocara* and *Toxoplasma* being more common in Korea) [15], *Bartonella* remains the leading cause of neuroretinitis reported in Southeast Asian populations. In a retrospective review of Malay patients with ocular bartonellosis, most patients did not report a history of cat bites or scratches, highlighting the importance of serological testing [16]. However, the low sensitivity of serological assays can lead to false negatives. As such, negative *Bartonella* serologies are insufficient to rule out CSD [17]. Repeat serologies within 10 to 21 days can help confirm a diagnosis. In our patient, the IgG titres increased from <1:64 to 1:128 over one week (albeit still equivocal), suggesting that the initial samples might have been obtained early in the course of the infection, before seroconversion.

While serological assays are the most used diagnostic tool, late seroconversion, cross-reactivity, and a lack of IgM in active infection limit the sensitivity of serology [18,19]. Reliable confirmatory tests for CSD have been sparse. Molecular diagnostics have been used to diagnose CSD with variable sensitivity, depending on the method of tissue sampling [20]. Recently, *Bartonella* neuroretinitis has been diagnosed using metagenomic next-generation sequencing with intraocular fluid samples [21]. However, in the evaluation of optic disc swelling of an unknown aetiology, intraocular fluid sampling may introduce unnecessary procedural risks. Given the self-limiting nature of CSD and *Bartonella* neuroretinitis, the utility of confirmatory testing might be called into question. However, CSD can also mimic demyelinating disorders [22], various causes of optic neuropathy (e.g., ischaemic optic neuropathy, papillitis, papilloedema) [14], and even haematological malignancies (e.g., lymphoproliferative disorders) [23,24,25], thus necessitating a degree of diagnostic certainty in its management. Supporting clinical features of CSD include fever and tender lymphadenopathy, which is present in the majority of cases [26]. A careful and targeted review of systems and systemic examination is, therefore, crucial, particularly in resource-limited practices, where serological testing may not be available [15].

The visual prognosis of *Bartonella* neuroretinitis is generally favourable, with many achieving a final BCVA of 6/12. Cases of counting-finger vision and absent RAPD have been reported, suggesting that serous detachment of the macula causes vision loss rather than optic nerve dysfunction [7]. In our patient, the spontaneous improvement in the BCVA accompanied the appearance of the macular star, a phenomenon which occurs when lipid-rich exudates penetrate the outer plexiform layer. Occasionally, symptom onset may precede macular star formation by 1 to 2 weeks [27]. In our patient, the macular star appeared 5 days after symptom onset. Serial dilated fundus examinations are, thus, crucial in the evaluation of suspected neuroretinitis to avoid missing a macular star.

Given its favourable natural history, evaluating treatment outcomes in neuroretinitis is challenging. Corticosteroids can be given as adjunctive therapy to reduce inflammation [28]. However, the mainstay of treatment depends on the aetiology. Broad-spectrum empirical antibiotics are appropriate while infective serologies are pending. Reed et al. reported a hastened visual recovery and a shortened disease course of *Bartonella* neuroretinitis with doxycycline and rifampicin [29]. In a retrospective study of 268 cases of CSD, rifampicin, ciprofloxacin, and trimethoprim-sulfamethoxazole were the most effective in improving the systemic symptoms (e.g., fever and headache) [30]. However, given CSD’s self-limiting nature and the high degree of spontaneous visual recovery in neuroretinitis, antibiotic efficacy is inconclusive at best.

## 4. Conclusions

This report highlights the fact that CSD remains an important differential in the evaluation of optic disc swelling. A thorough systems review should be sought in all patients presenting with sudden-onset painless vision loss. A careful and targeted systemic examination is crucial when suspecting CSD (fever, tender lymphadenopathy), as serological testing may be unavailable or, as in our case, falsely negative. The macular star sign may not be present in the early stages of neuroretinitis, but subretinal and intraretinal fluid can be reliably demonstrated via OCT in the early stages of the disease, highlighting the importance of serial dilated fundus examination and early referral to an ophthalmologist, which can help streamline disease management. Negative serologies are insufficient in ruling out CSD, and repeat assays in the later stages of the disease can be helpful in guiding its management.

## Figures and Tables

**Table 1:** Causes of optic disc swelling (* generally, the presence of a macular star precludes a demyelinating aetiology).

Localisation	Intracranial	Retrobulbar	Ocular (Retina, Choroid, Optic Nerve Head)
**Causes**	Space occupying lesion Malignant hypertension Venous sinus thrombosis Idiopathic intracranial hypertension Meningitis	Inflammatory/demyelinating (e.g., optic neuritis, neuroretinitis) Ischaemic Compressive Others (e.g., traumatic, nutritional, hereditary)	Central retinal vein occlusion Infiltration Inflammatory (e.g., neuroretinitis, uveitis) Hypotony (decompression)
**Symptoms**	Headache Nausea and vomiting Diplopia Transient vision loss	Blurring of vision (typically progressive) Loss of colour vision Pain on eye movement	Blurring of vision, floaters Eye pain/redness
**Ocular signs**	Unilateral/bilateral disc swelling False localising sixth nerve palsy Macular star *	Reduced colour vision Relative afferent pupillary defect (if asymmetrical) Proptosis	Redness Cells in anterior chamber, vitritis Retinal vasculitis Retinal haemorrhages Macular star *
**Systemic signs**	Raised blood pressure Neck stiffness Fever	Focal neurological signs (e.g., numbness, weakness) Normal examination	Cervical lymphadenopathy Skin changes

## Data Availability

Data will be made available upon request.

## Abbreviations

BCVA: best corrected visual acuity; CSD: cat-scratch disease; FFA: fundus fluorescein angiography; IgG: immunoglobulin G; MOGAD: myelin oligodendrocyte glycoprotein antibody-associated disease; MRI: magnetic resonance imaging; NMOSD: neuromyelitis optica spectrum disease; OCT: optical coherence tomography; and RAPD: relative afferent pupillary defect.
